# Analyzing Person-Place Interactions During Walking Episodes: Innovative Ambulatory Assessment Approach of Walking-Triggered e-Diaries

**DOI:** 10.2196/39322

**Published:** 2022-11-25

**Authors:** Martina Kanning, Lukas Bollenbach, Julian Schmitz, Christina Niermann, Stefan Fina

**Affiliations:** 1 Department of Sport Science University of Konstanz Konstanz Germany; 2 Faculty of Architecture and Civil Engineering University of Applied Sciences Augsburg Augsburg Germany; 3 Institute of Interdisciplinary Exercise Science and Sports Medicine Medical School Hamburg Hamburg Germany

**Keywords:** ecological momentary assessment, active transport, socio-ecological model, subjective well-being, mental health, urban health, GEMA, geographically explicit ecological momentary assessment, behaviour change, walking, experience, environment, monitoring, activity, tracking, e-diary, assessment

## Abstract

**Background:**

Walking behavior is positively associated with physiological and mental health as much evidence has already shown. Walking is also becoming a critical issue for health promotion in urban environments as it is the most often used form of active mobility and helps to replace carbon dioxide emissions from motorized forms of transport. It therefore contributes to mitigate the negative effects of climate change and heat islands within cities. However, to promote walking among urban dwellers and to utilize its health-enhancing potential, we need to know more about the way in which physical and social environments shape individual experiences during walking episodes. Such person-place interactions could not adequately be analyzed in former studies owing to methodological constraints.

**Objective:**

This study introduces walking-triggered e-diaries as an innovative ambulatory assessment approach for time-varying associations, and investigates its accuracy with 2 different validation strategies.

**Methods:**

The walking trigger consists of a combination of movement acceleration via an accelerometer and mobile positioning of the cellphone via GPS and transmission towers to track walking activities. The trigger starts an e-diary whenever a movement acceleration exceeds a predetermined threshold and participants' locations are identified as nonstationary outside a predefined place of residence. Every 420 (±300) seconds, repeated e-diaries were prompted as long as the trigger conditions were met. Data were assessed on 10 consecutive days. First, to investigate accuracy, we reconstructed walking routes and calculated a percentage score for all triggered prompts in relation to all walking routes where a prompt could have been triggered. Then, to provide data about its specificity, we used momentary self-reports and objectively assessed movement behavior to describe activity levels before the trigger prompted an e-diary.

**Results:**

Data of 67 participants could be analyzed and the walking trigger led to 3283 e-diary prompts, from which 2258 (68.8%) were answered. Regarding accuracy, the walking trigger prompted an e-diary on 732 of 842 (86.9%) reconstructed walking routes. Further, in 838 of 1206 (69.5%) triggered e-diaries, participants self-reported that they were currently walking outdoors. Steps and acceleration movement was higher during these self-reported walking episodes than when participants denied walking outdoors (steps: 106 vs 32; acceleration>0.2 *g* in 58.4% vs 19% of these situations).

**Conclusions:**

Accuracy analysis revealed that walking-triggered e-diaries are suitable to collect different data of individuals' current experiences in situations in which a person walks outdoors. Combined with environmental data, such an approach increases knowledge about person-place interactions and provides the possibility to gain knowledge about user preferences for health-enhancing urban environments. From a methodological viewpoint, however, specificity analysis showed how changes in trigger conditions (eg, increasing the threshold for movement acceleration) lead to changes in accuracy.

## Introduction

### Background

Walking with low to moderate intensity is associated with physiological health such as all-cause and cardiovascular mortality [1], obesity and diabetes [2], and mental health disorders such as stress [3], depression [4,5] and well-being [6-8]. Besides these health-enhancing associations, walking is the most often used type of active mobility [9]. Walking as well as active mobility replace carbon dioxide emissions from motorized transport and therefore contribute toward mitigating anthropogenic climate warming [10]. Such health and climate effects are of particular concern for urban residents who are exposed to higher levels of stress, isolation, and air pollutants and emissions [11-13]. Thus, increasing walking episodes and active mobility are important objectives for health promotion in urban environments [14,15]. However, to promote walking among urban dwellers in their everyday life and to utilize its health-enhancing potential, we need to know more about the way physical and social environments shape individual experiences during walking episodes. It is important to understand how a resident reacts to contextual circumstances (eg, street greenery and wide sidewalks) and which person or situation-specific moderators (eg, lifestyle, attitude, social interaction, and weather or noise condition) affect the association between walking outdoors and health [16,17]. Such knowledge would help to inform the development of urban designs, which are associated with positive effects while walking in daily urban life.

To analyze such person-place interactions between the individuum within its environment, we need time-varying associations, which previous studies mostly do not provide. The goal of this study is to introduce walking-triggered e-diaries as an innovative ambulatory assessment approach to overcome this methodological constraint.

### Assessing and Analyzing the Health-Enhancing Potentials of Urban Environments

A plethora of studies and reviews investigated associations between the built or physical environment and physical activity (eg, walking). They indicated that high-quality walking infrastructure, access to recreational facilities, as well as parks, trails, or new infrastructure for walking are associated with increased levels of physical activity [18,19]. Regarding the impact on mental health, the findings are not as consistent as they are for physical activity but show that park-based and greenway interventions can promote improved mental health [20,21]. However, these findings are mostly based on cross-sectional or longitudinal data with longer time frames, and they rarely examine moderating effects of the environment in a way that allows investigation of person-place interactions. For this, an ambulatory assessment (AA) is a useful approach [22,23]; it allows researchers to examine time-varying associations of, for example, momentary affective states and physical activity and to what extent environmental features in that specific situation moderate this association. Hereinafter, we use the term “AA” instead of the “experience sampling method” or “ecological momentary assessment” (EMA), as it covers the daily assessment of self-reports (via paper-and-pencil or electronic diaries) in combination with the monitoring of physiological functions (eg, heart rate and electrodermal activity), behavior (eg, via accelerometers and mobile electrocardiograms), or environmental parameters (eg, via geolocation tracking) [24]. Several advantages, such as assessments in everyday life, in real time, and repeated measurements with a high sampling frequency led to the use of AA in a wide range of research areas [25]. Two general categories of data sampling are usually distinguished: (1) time-based designs and (2) event-based designs. To capture variability of a certain health or psychosocial outcome (eg, affective states and different kinds of social interactions), a time-based design is appropriate for recording data at fixed (eg, every hour) or random intervals. However, when the aim is to extract data in specific situations in everyday life (eg, walking outdoors), event-based designs are recommended. This design requires the participants to detect preselected situations on their own and to provide their self-reports during or after such situations. However, technical progress of accelerometers and sensors in recent years allow for triggers as a sophisticated feature within an event-based design. Sensors (eg, accelerometers and GPS) can discover in situ a predefined situation in everyday life and initiate an alarm to answer e-diaries. This triggered event–based approach minimizes barriers for participants and increases the objectivity of self-reporting when this predefined event occurs (for an overview, see Shiffman et al [26]).

### Technical Progress in Assessing Person-Place Interactions

In recent years, some research groups in the field of urban development and city planning extended assessment ideas and collected momentary data of mental health or subjective environmental experiences during everyday life. These study designs combined mobile geographic location technologies (eg, GPS) to assess precisely the space where participants spend their time outdoors. Time-based assessments via daily diaries resulted in geographically explicit ecological momentary assessments (GEMA; for an overview, see Chaix [16] and Kirchner et al [27]). As an example, a GEMA-study assessed the association between urban green space and stress in 13- to 14-year-old adolescents living in urban surroundings [28]. Participants were recruited from an adolescent medicine outpatient clinic. Momentary experiences of stress were captured 3-6 times a day over a 4-day period every second month over a 2-year period. Participants were equipped with a GPS-enabled mobile phone. They received text messages including a link to a web-based questionnaire with a single item measuring momentary stress. Exposure to greenspace was calculated retrospectively for each participant using the normalized difference vegetation index of all image pixels within 100 m of each EMA location. This index measures leaf abundance in green vegetation. The analyses included 179 participants providing 9346 EMA responses and revealed significant associations between exposure to green space and lower psychological stress during active mobility (see also the feasibility study of GEMA by Boettner et al [29] with a representative sample of 1405 adolescents living in cities and suburban municipalities).

Electronic diaries within GEMA designs therefore allow capturing of subjective experiences in situ. The data can be linked retrospectively to participants’ current position in time and space. However, not all GEMA studies explicitly assessed physical activity (eg, with accelerometers) but rather operationalized activity using time-space patterns of the GPS. In addition, most GEMA designs struggled with the disadvantages of “time-based” assessments because prompts to answer the web-based questionnaires were usually triggered at regular time intervals. With such a sampling scheme, self-reports during rare events (eg, walking episodes) are likely to be missed, which impedes the analysis of person-place interactions. A currently published study protocol of a GEMA study attempted to overcome this restriction (Fernandes et al 2020 [30]). This GEMA study included older residents of Paris (>60 years old) and aimed to assess whether the sequence of stressful environmental exposures in everyday life explained within-subject variability in stress and depression. They presented a novel methodology combining GEMA with an algorithm (based on the smartphone’s built-in GPS receiver) that allowed triggering of EMA surveys only when subjects are outdoors. However, the trigger did not differentiate between active and inactive episodes and is therefore not able to identify walking episodes.

The purpose of this study is to introduce walking-triggered e-diaries as an innovative ambulatory assessment approach to overcome the methodological shortcomings mentioned above. It combines movement acceleration, time, and distance assessments (GPS) to trigger an e-diary. The aim of this study is to examine its accuracy through 2 different approaches. First, we reconstructed walking routes (using a GPS) and examined on how many walking routes the walking trigger prompted an e-diary (sensitivity). Second, we describe activity levels (movement acceleration and number of steps) immediately before the prompt, and used self-reports to identify “false positive” walking-triggered e-diaries; that is, a participant received a triggered e-diary, but denied walking outdoors (specificity).

## Methods

### Walking-Triggered e-Diaries: How Do They Work

Triggered e-diaries (or triggered EMAs) have previously been employed to assess data during physically active and inactive episodes [31], as well as during prolonged sedentary bouts [32] (see also the comparison of different GPS-based triggered e-diaries by Törnros et al [33]). To develop the walking-trigger, we used the following equipment: a hip-worn accelerometer (Move3, movisens.com), a smartphone (Motorola, running on the Android operating system) to show e-diaries (movisensXS), and location tracking via GPS and transmission towers, combined in a technical interface between the smartphone and the accelerometer (via Bluetooth low energy). Move3 captured the body position and movement acceleration within a range of ±16 *g* and with a sampling frequency of 64 Hz. Move3 has been validated for documenting movement acceleration and different body positions [31,34].

Taken together, the walking trigger consists of a combination of movement-acceleration and mobile positioning of the cellphone via GPS and transmission towers to identify walking episodes. The walking trigger prompts an e-diary between 6 AM and10 PM whenever an individual is walking outdoors. This is achieved by distinguishing between 2 states: stationary and nonstationary. The trigger state switches to nonstationary whenever a movement acceleration exceeds a predetermined threshold (>0.1 *g* for at least 1 minute) and the participants’ location changes beyond a radius of 100 m. If these conditions are met, a walking-triggered e-diary (main) is prompted. Furthermore, participants receive follow-up walking-triggered e-diaries on their walking trip with a repeat interval of 420 seconds and a randomization time of 300 seconds for as long as the trigger conditions are met (repeated). For each e-dairy, participants need approximately 1 minute for responding.

To conduct the walking-triggered e-diary study, the trigger sampling scheme and the corresponding forms in the movisensXS browser app [35] have to be created. Next, the technical interface, consisting of a study smartphone (which depicts the e-diaries and enables tracking via GPS and transmission towers) and the Move3 accelerometer, was established via Bluetooth low energy. Finally, the study was prepared to be started by the participants once they received their smartphone and accelerometer.

### Data Processing

The collected data were processed with software (UnisensViewer, DataAnalyzer, and DataMerger) available at the manufacturer’s website home page [36]. All data forms from the smartphones were then uploaded to the movisensXS browser app. Corresponding data from the accelerometers were extracted with the manufacturer’s data software, DataAnalyzer (versions 1.13.5 and 1.13.7). Next, physical activity was calculated in 1-minute intervals for better interpretation. All e-diary and smartphone data from the movisensXS browser app were then downloaded to synchronize and merge them with the accelerometer data, using the manufacturer’s software DataMerger (version 1.8.0). After the successful combination of all relevant data, we applied predetermined validity criteria of the data, regarding 8-hour wear-time, location accuracy, and answered prompts.

In a final step, spatial information and data on environmental features (eg, greenness and noise pollution) were added for further analyses. Data fusion of the e-diary and accelerometer data with GIS information require that GPS data are linked to the concomitant e-diary via a unique ID composed of the participant ID and the time stamp of the e-diary or accelerometer data.

### Participant Recruitment and Study Procedure

For participant recruitment, 3000 letters were distributed to preselected urban households with an invitation to participate in a web-based, cross-sectional questionnaire. The data collected in the survey were used for purposes not emphasized in this study, except for the final step of the web-based questionnaire. Individuals, who want to and who fit the inclusion criteria could join the walking-triggered e-diary assessment. Inclusion criteria were for individuals to understand the German language (as the questionnaires were in German), to be older than 18 years, to live in an urban or suburban area, and to have no mental or physical health conditions that would restrain them from being physically active (eg, injuries or depression). Furthermore, participants were offered a profile of their activity pattern during the duration of the assessment and an incentive of €50 (US $51.86) if they provide data on at least 7 days. Potential participants received information regarding the study and the procedure and instructions on how to wear and use the sensors and smartphones before written consent was obtained. In addition, a video was provided with the same information for further clarification.

After a confirmatory phone call, participants were equipped with a smartphone and a sensor via a mailed package. In the cases where no questions came up, the participants were able to start the study by themselves. Participants were consecutively recruited from July to December 2020 (no lockdown conditions during this time owing to COVID-19 restrictions in Germany). The study period per participant lasted 10 consecutive days with an option to extend the study voluntarily. During the assessed time period, between 6 AM and 10 PM, participants received walking-triggered prompts, requesting them to answer short e-diaries that were shown on their smartphones. In addition, they randomly received 3 further e-diaries over the course of the day (between 10 AM and 10 PM, with a minimum 2.5-hour interval in between). After completing the study, the participants returned the smartphone-sensor combination via prepaid mail packages.

### Ethical Considerations

The study received full ethical approval from the University of Konstanz (IRB18KN010-004; October 29, 2018).

### Statistical Analyses

Two different approaches were used to evaluate the accuracy of the walking trigger algorithm. First, we assessed accordance between walking routes and walking-triggered e-diaries (sensitivity). To reconstruct as many walking routes as possible, we used all recorded GPS signals, with the following conditions: GPS signals with a time span of 60-120 seconds, each GPS-signal has an accuracy of at least 30 m, mean speed of the total route is less than 10 km/hour, and a minimum walking distance of 100 m.

**Figure 1:**
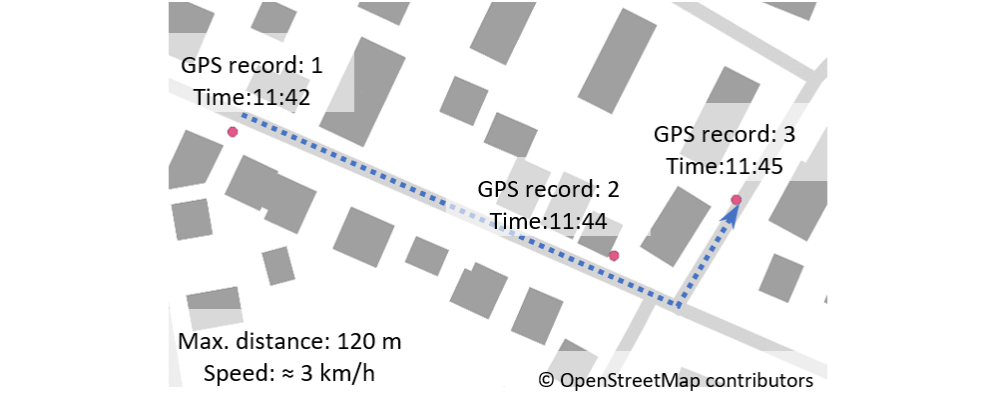
Survey locations along the walking routes (adapted from ©OpenStreetMap, licensed under the Open Data Commons Open Database License [37]).

We modeled the walking routes with the statistical analysis software R and the OpenRouteService routing system (HeiGIT gGmbH). The reconstructed walking routes are calculated independently from the walking-triggered e-diaries and were used as reference data. We checked for how many walking routes were e-diaries triggered (Figure 1).

Furthermore, we compared how many trigger-based versus time-based prompts started an e-diary during a walking route to give an impression of the added value of using the walking-triggered approach instead of a time-based approach. In the next step, we focused on the specificity of the walking trigger approach and verified whether a participant was walking outside immediately before a walking-triggered e-diary was prompted (only main e-diaries were used). For this purpose, we used the self-reports of the main e-diaries. In addition, we compared steps and accelerometer data before main e-diaries were triggered between situations in which participants self-reported that they were or were not walking outdoors.

## Results

### Descriptive Statistics

We could analyze data of 67 of 79 recruited participants (excluded participants, n=12; technical problems, n=4; wear time<8 hours, n=7; opted out of the study, n=1). The excluded participants (n=12) did not differ significantly in age (mean 40.4, SD 13 years) and sex (sex distribution of the excluded participants was 50% each for women and men) from the included participants. The included participants (N=67) had a mean age of 41.0 (SD 14.6) years, and 50.7% were female. 82.1% of participants had a high school certificate, and the average BMI was 23.9 (SD 3.8). Participants provided a total of 632 days of sensor data (mean per participant 9.4, SD 1.8, range 3-12 days; minimum wear time of 8 hours per day; mean wear time per participant per day of 11 hours 59 minutes, SD 2 hours 46 minutes). At least 1 e-diary was triggered on 452 days, which yielded an average of 6.7 (SD 2.9) diaries per day. The walking-trigger produced 3283 e-diary prompts within the study period (mean per participant 49, SD 44.3, range 1-179). Of a total of 3283 prompts, 2258 (68.8%) were answered, including 1206 of 1840 main e-diaries (mean per participant per day 2.8, SD 2.1, range 1-20) and 1052 of 1443 repeated e-diaries (mean per participant per day 2.5, SD 1.5, range 1-21). The resulting compliance rates were 65.5% (1206/1840; main e-diary) and 72.9% (1052/1443; repeated e-diary), respectively. In addition, participants received 1955 randomly prompted e-dairies (mean per participant per day 2.9, SD 0.1, range 1-3). Of a total of 1955 randomly prompted e-diaries, 1479 (75.6%) were completed (mean per participant 22.1, SD 7.1, range 1-35).

### Did the Walking Trigger Algorithm Trigger an e-Diary During Walking Routes (Sensitivity)?

**Figure 2:**
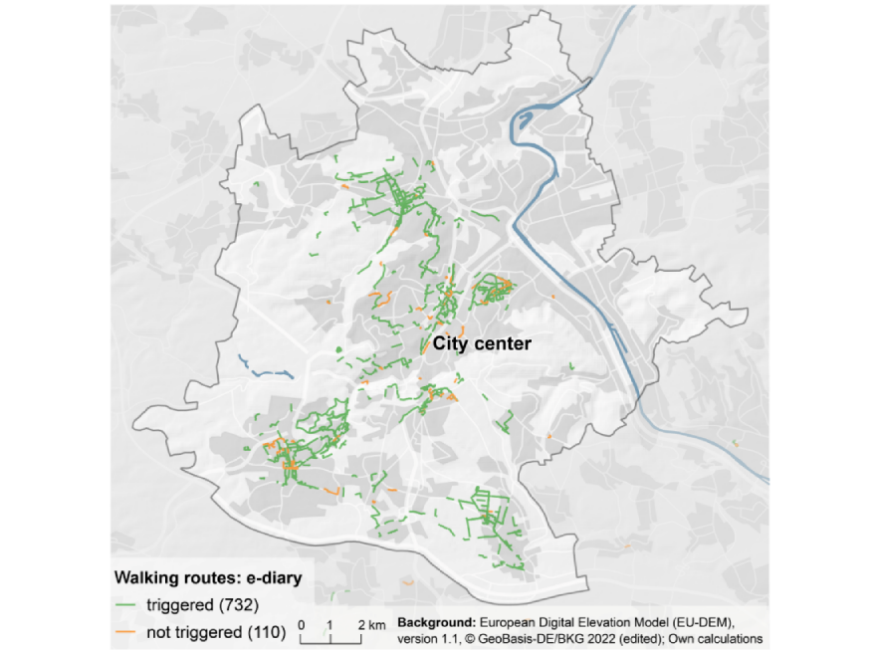
Walking routes in Stuttgart identified via GPS (adapted from ©GeoBasis-DE / BKG (2020) [38], licensed under Data licence Germany – attribution – Version 2.0 [39] and Copernicus Services that delivers data with funding by the European Union [40]). Note: the authors used their own color scheme and calculations.

We reconstructed a total of 842 walking routes from recorded GPS signals. In 732 situations, at least 1 e-diary (*main* or *main and repeated*) was triggered during or immediately after a walking route (maximum 2 minutes). This yielded an overall accuracy of 86.9% (732/842; Figure 2). Using a time-based trigger (3 times randomly throughout the day), which was often used in previous GEMA studies, led to an e-diary during walking episodes in only 29 of 842 (3.4%) situations.

### Did the Participants Walk Outdoors Immediately Before a Main e-Diary Was Triggered (Specificity)?

**Figure 3:**
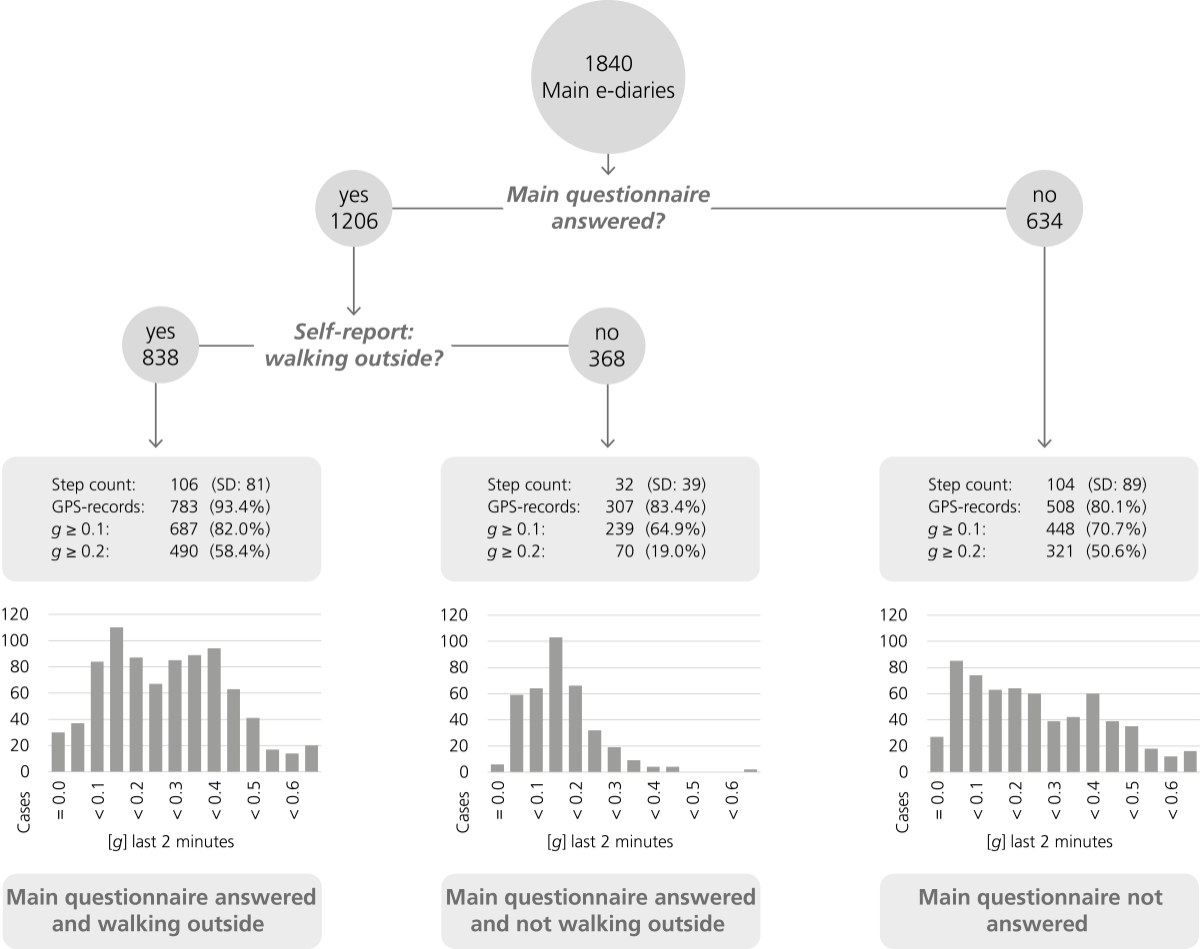
Comparison of activity level (step count and movement acceleration in *g*) between situations in which participants self-reported walking or not walking outdoors and unanswered situations.

In total, 1840 main e-diaries were triggered, of which 1206 were answered. In 838 of the 1206 (69.5%) situations, the participants indicated via self-report that they were (currently) walking outdoors (Figure 3).

In these 838 situations, an average of 106 (SD 81) steps were taken in the 2 minutes before the e-dairy, and the acceleration was >0.1 *g* in 82.0% and ≥0.2 *g* in 58.4% of these situations. GPS records were present 783 out of 838 (93.4%) times. However, in 368 of the 1206 (30.5%) situations, participants indicated via self-report that they were not (currently) walking outdoors. In the 2 minutes before these triggered e-diaries, an average of 32 (SD 39) steps were taken, and in 64.9% and 19.0% of cases, the acceleration was >0.1 *g* and ≥0.2 *g*, respectively. GPS records were present 307 times (307/368, 83.4%). Unanswered main questionnaires amounted to a total of 634. The following movement data for these situations can be reported: an average of 104 (SD 89) steps were recorded in these situations, and in 70.7% and 50.6% of cases, the acceleration was at least 0.1 *g* and ≥0.2 *g*, respectively. GPS records were present 508 out of 634 (80.1%) times.

## Discussion

### Principal Findings

This study introduces walking-triggered e-diaries as an innovative measurement approach for walking behavior and evaluates its accuracy for this purpose. This new method allows for the analysis of time-varying associations to deepen our understanding of person-place interactions. It can potentially assess data of the person (eg, mental health criteria including momentary affective states) as well as the social and physical environment (eg, interacting with others and experiencing greenness) during the very situations in which a person walks outdoors.

According to the results of the reconstructed walking routes about sensitivity, in 86.9% of these walking episodes, participants received a triggered e-diary. This finding shows that walking-triggered e-diaries had high accuracy in capturing data during episodes of walking outdoors. Compared to a time-based assessment, which was usually used in previous studies (eg, GEMA studies), the walking trigger is more sensitive and leads to a relevant higher number of e-diaries during episodes of walking outdoors (732 vs 29, respectively). Nevertheless, there are some reconstructed walking routes where participants did not receive a triggered e-diary. As the trigger depends on movement acceleration and not explicitly on gait velocity, it is possible that the participant's movement acceleration had been lower than the set threshold value of 0.1 *g*. Detection of gait velocity or inclusion of steps in the algorithm of the walking trigger potentially mitigate this inaccuracy.

The assessment approach of walking-triggered e-diaries extends former studies, which have already shown that, for instance, more green space during active episodes is associated with lower stress levels [28]. However, to improve our knowledge about environmental effects on participants’ behavioral outcomes and mental health, relevant constructs and variables should be assessed in situ, precisely in the situation during which a person experienced his or her environment [17,41]. With a time-based assessment (eg, e-diaries were prompted 4 times a day), it is difficult to explicitly capture episodes of walking outdoors. For instance, former GEMA studies showed that almost more than two-thirds of their GEMA responses occurred at home and not during episodes of walking outdoors (eg, 72% of home responses) [28]. This high number of home responses instead of assessments during outdoor walking episodes was to be expected in time-based assessments, as physically active episodes are rare during everyday life; this is because most people worldwide are insufficiently physically active and do not meet the recommended activity level of 150 minutes of moderate activity per week [42]. Calculated at 12 hours for 1 day of assessment, most people only use 3% of their time for physical activity. It is therefore highly probable that time-based assessments miss some episodes of walking outdoors. Walking-triggered e-diaries enhance the possibility to assess data during episodes of walking outdoors.

The second analysis was related to the specificity of the walking trigger assessment. It used participants' self-reports to provide an impression of false positive triggered e-diaries and used movement acceleration and step counts to estimate activity levels immediately before the e-diary was triggered. However, participants reported, in 30.5% of the walking-triggered e-diaries, that they were currently not walking outdoors. According to the objectively measured activity data during these situations, there had been sufficient movement acceleration, and the accelerometer identified on average 32 steps per person during the last 2 minutes immediately before the prompt. Thus, owing to the objectively measured activity levels, the walking trigger algorithm’s prompt was accurate. Participants, however, did not actively indicate these movements as outdoor walking behavior. A possible explanation is that a person was strolling around his or her home or performed some leisure time activities such as slow roller-skating or slow bicycling. Such activities can lead to a nonstationary movement acceleration greater than 0.1 *g* (as GPS and acceleration condition is true by value), but the person did not interpret the movement as walking outdoors. Overall, our findings emphasize the importance of assessing participants' interpretations and behavior patterns apart from objective measures of physical activity. Furthermore, the histograms in Figure 3 provide an impression of how the movement acceleration of physical activity is related to self-reported (non-) stationary active episodes. When participants denied that they were engaged in walking outdoors, acceleration was mostly lower (19%; >0.2 *g*; 32 steps) than that in situations where participants confirmed activity (58.4%; >0.2 *g*; 106 steps). This observation leads to the conclusion that an increase in the activity threshold to 0.2 *g* of movement acceleration would help reduce false positive e-diaries but would also reduce sensitivity (ie, fewer walking episodes will be identified).

Our study design with walking-triggered e-diaries reveals acceptable compliance rates of 65% for the triggered main e-diaries and 72% for the repeated ones. These numbers are in line with those of other studies assessing walking triggers [43]. However, the compliance rate is difficult to compare because it depends heavily on the study design and the burden participants have with providing self-reports several times per day for several consecutive days [44].

Although the data set included 632 days of sensor data, at least 1 walking-triggered e-diary was triggered on only 452 days. We therefore have 180 days of no data on outdoor walking activity (an average of 2.7 days per participant during a 10-day assessment period). This number seems quite high. Part of the explanation is, however, that data assessments have been conducted during a time where COVID-19 restrictions imposed heavy constraints on participants to meet other people, and working from home was recommended or even mandatory for some employees. Several studies showed that physical activity levels and walking activities were reduced during this time [45].

### Strengths, Limitations, and Implications for Future Studies

The strength of our new measurement approach of walking-triggered e-diaries is 2-fold: first, it incorporates accelerometers to assess physical activity directly and combines activity level and mobile positioning of the cellphone via GPS and transmission towers to identify walking outdoors. Second, it uses these identified outdoor walking episodes to start an e-diary. Such a design expanded existing activity-triggered assessments because it can differentiate between indoor activities or stationary outdoor activities and walking behavior. Thus, person-place interactions with the physical and social environment, participants’ walking behavior, and how these constructs might moderate health-enhancing effects can be examined exactly in those situations in which a person is walking outdoors. This design also minimizes the burden for participants, since they receive questionnaires predominantly in situations in which a relevant behavior occurs. In addition, this study highlights the importance of assessing participants’ interpretation of their current behavior when assessing and analyzing person-place interactions.

Nevertheless, walking-triggered e-diaries do face some challenges. First, the topography of cities limits the accuracy of GPS signals because buildings restrict their transmission to satellites [46]. Second, participants had to wear an extra sensor (accelerometer) and were equipped with a smartphone for the study. To increase the compliance rate and reduce missing data, technical solutions in which participants install an app on their own smartphone would be preferable. Third, expertise in data handling is an essential precondition for processing (eg, movement acceleration, GPS signals, and merging of different data sets) and analyzing (eg, multilevel modeling) the results. It requires interdisciplinary competencies (eg, spatial science, sport and movement science, and psychology) to facilitate such studies in the field of urban health.

In future studies, ambulatory assessments with walking-triggered e-diaries could be enriched with further sensor-based data (eg, momentary noise or heart rate and electrodermal activity) to enhance knowledge about different momentary exposures, experiences, and behavior during active mobility [17]. Furthermore, walking-triggered e-diaries can be used to develop just-in-time adaptive intervention by delivering support (eg, feedback prompts) when users need them most (eg, encourage to walk for a longer distance or start to walk after a longer episode of inactivity) [35].

### Conclusions

This study presents an accuracy analysis for a new method in ambulatory assessments. Our results reveal that walking-triggered e-diaries are suitable to collect different data of individuals' current experiences (eg, affective states, social interactions, and environmental features) during episodes of walking outdoors. Analyzing time-varying data during these predefined situations provides new insights into person-place interactions; that is, how physical and social environments shape individual experiences when walking outdoors. Based on the increasing interest in the design of health-promoting urban environments in recent years, such a design can offer substantial methodological progress. However, analyzing person-place interactions requires expert knowledge on data handling (eg, GPS and walking routes, movement acceleration, and e-diaries) and the management of intensive longitudinal studies in interdisciplinary teams.

## Abbreviations

AA: ambulatory assessment
EMA: ecological momentary assessment
GEMA: geographically-explicit ecological momentary assessment
